# Microsoft HoloLens 2 in Medical and Healthcare Context: State of the Art and Future Prospects

**DOI:** 10.3390/s22207709

**Published:** 2022-10-11

**Authors:** Arrigo Palumbo

**Affiliations:** Department of Medical and Surgical Sciences, Magna Græcia University, 88100 Catanzaro, Italy; palumbo@unicz.it

**Keywords:** HoloLens, head-mounted display, augmented reality, mixed reality, virtual reality, telemedicine, remote control

## Abstract

In the world reference context, although virtual reality, augmented reality and mixed reality have been emerging methodologies for several years, only today technological and scientific advances have made them suitable to revolutionize clinical care and medical contexts through the provision of enhanced functionalities and improved health services. This systematic review provides the state-of-the-art applications of the Microsoft^®^ HoloLens 2 in a medical and healthcare context. Focusing on the potential that this technology has in providing digitally supported clinical care, also but not only in relation to the COVID-19 pandemic, studies that proved the applicability and feasibility of HoloLens 2 in a medical and healthcare scenario were considered. The review presents a thorough examination of the different studies conducted since 2019, focusing on HoloLens 2 medical sub-field applications, device functionalities provided to users, software/platform/framework used, as well as the study validation. The results provided in this paper could highlight the potential and limitations of the HoloLens 2-based innovative solutions and bring focus to emerging research topics, such as telemedicine, remote control and motor rehabilitation.

## 1. Introduction

Virtual reality (VR), augmented reality (AR) and mixed reality (MR) have been emerging methodologies for several years, but only today technological and scientific advances have made them suitable to allow users to experience a spectacular imaginary world, generating realistic images, sounds and other sensations [1].

Although VR, AR and MR may seem apparently similar terms, it is necessary to deepen their definition in order to differentiate their functioning. 

VR, the most widely known technology, is completely immersive and deceives the senses into thinking that you are in a different environment or in a parallel world compared to the real one. In a virtual reality environment, using a head-mounted display (HMD) or headset, a user feels completely immersed in an alternate reality and can manipulate objects while experiencing computer-generated visual effects and sounds. 

Alternatively, augmented reality [2] is characterized by the ability to overlay digital information on real elements. Augmented reality keeps the real world at the center, but enhances it with other digital details, bringing new layers of perception and complementing reality or environment. 

Mixed reality [3] blends elements of the real and digital worlds. In mixed reality, the user can interact and move elements and environments, both physical and virtual, using the latest generation of sensory and imaging technologies. It offers the possibility of having one foot (or one hand) in the real world and the other in an imaginary place, breaking down the basic concepts of reality and imagination.

In the world reference context, the importance of VR, AR and MR technologies has been recognized in several fields (including healthcare, architecture and civil engineering, manufacturing, defense, tourism, automation and education) [1]. The wave of digital transformation has mainly involved the medical sectors, Indeed, the combination of the advanced digital platforms for handling big data and the high-performance viewing devices using a head-mounted display, has been definitely useful for diagnostics and treatment clinical decisions [4,5]

Scientific and technological advances in this area have enabled the design and development of several devices such as Google Glass, Vuzix Blade and Epson Moverio [6], making them suitable to revolutionize clinical care and medical contexts through the provision of enhanced functionalities and improved health services.

Microsoft^®^ HoloLens [7] was developed and manufactured by Microsoft (MS) and can be presented as a pair of mixed reality smart glasses able to describe an environment in which real and virtual elements appear to coexist. More specifically, the Microsoft^®^ HoloLens is a novel MR-based HMD that makes the user the protagonist of an immersive experience and allows him to interact with the surrounding environment using holograms whilst engaging their senses throughout. It is used in a variety of applications such as medical and surgical aids and systems, medical education and simulation, architecture and several engineering fields (civil, industrial etc.) [1].

The first generation of HoloLens [8], released in 2016, attracted the consideration of the scientific and technological context because of its advanced playing methods and concepts.

In November 2019, Microsoft Corporation released the subsequent HoloLens 2 [9], which is an upgrade in terms of hardware and software, compared with its predecessor. Indeed, to address the hardware and software limitations of the HoloLens version1, including its restricted field of view, limited battery life, and relatively heavy headset, Microsoft introduced the HoloLens 2, which presents an enhanced field of view (52°), reduced weight (566 g), and improved battery life (3 h) [10].

The rapid advancements in technology over the last decade has significantly impacted the medicine and health sciences. Driven by the growing need to make health care safer, the use of eXtended Reality (XR) (virtual, augmented, mixed) throughout the continuum of medical education and training is demonstrating appreciable benefit.

The epidemiological context of the coronavirus disease 2019 (COVID-19) pandemic is an unprecedented opportunity to speed up the development and implementation of innovative devices and biomedical solutions, as well as the adoption of eXtended Reality modalities that have experienced a tremendous increase in demand. Indeed, they played an important role in the fight of this pandemic through their deployment in various crucial areas such as telemedicine, online education and training, marketing and healthcare monitoring [11].

Remarkable advantages have already been identified from using the HoloLens for medical use [1], from training in anatomy and diagnostics to acute and critical patient care, such as for visualizing organs prior to surgery [12], teaching dental students [13], and in pathology education [14].

This paper aims to present the state-of-the-art applications of the Microsoft^®^ HoloLens 2 in a medical and healthcare context. Focusing on the potential that this technology has to revolutionize care, also but not only in relation to the COVID-19 pandemic, studies that proved the applicability and feasibility of HoloLens 2 in a medical and healthcare scenario were considered. 

The review presents a thorough examination of the different studies conducted since 2019, focusing on HoloLens 2 sub-field applications, device functionality as well as natively integrated or other integrative software used.

The rest of this paper is organized as follows: Section 2 details the methodology used for this review. Section 3 illustrates a synthetic overview of most popular commercially available optical see-through head-mounted displays and examines in depth the HoloLens 2 technical specifications and the comparison with the previous version (HoloLens 1), justifying the choice to focus our review only on the latest version. Section 4 summarizes the different existing solutions describing the HoloLens 2 applications in a medical and healthcare context. Section 5 discusses the current status and trends in research based on HoloLens 2 by year, subfield, type of visualization technology, device functionalities provided to users and software/platform/framework used, as well as study validation, while Section 6 concludes the study, focusing on the potential of this innovative technology in a biomedical scenario.

## 2. Research Methodology

### 2.1. Search Strategy

This systematic review was conducted following the preferred reporting items for systematic reviews and meta-analyses [15]. A comprehensive literature search was conducted on 19 April 2022. The most common engineering and medical databases (IEEE Xplore, PubMed, Science Direct and Scopus) were selected for research, as reported in Table 1. The review was limited to texts published in English between 2019 and 2022, and for which abstracts were available. Considering the scope of the systematic review, the specific keywords were defined. This structured search string was used to organize this paper: “HoloLens 2” OR “MS HoloLens 2”—AND—“Healthcare” OR “Medicine”. In addition, the articles identified through the reference list of previously retrieved articles were included in order to increase the likelihood that all the relevant studies were identified.

### 2.2. Inclusion and Exclusion Criteria

Articles were considered for inclusion only if: (1) they used at least the Microsoft^®^ HoloLens version 2 (studies that described a comparison between first and second generation HoloLens were also considered) but not exclusively version 1; (2) they described a partial or total demonstration of the feasibility, effectiveness, and applicability of HoloLens v.2 in a medical and healthcare context; (3) they described complete research; (4) they are written in the English language.

The articles were also screened for the following exclusion criteria: (1) contributions in which the information related to the HoloLens version is not specifically reported; (2) studies that described application of HoloLens 2 in studies involving animals and not human; (3) HoloLens 2 application field is different from medical or healthcare context; (4) articles without full-text available.

Exclusion criteria were also related to books or book chapters, letters, review articles, editorials, and short communications.

### 2.3. Study Selection

The state-of-the-art applications of MS HoloLens 2 in a medical and healthcare context is presented in this review. 

**Figure 1:**
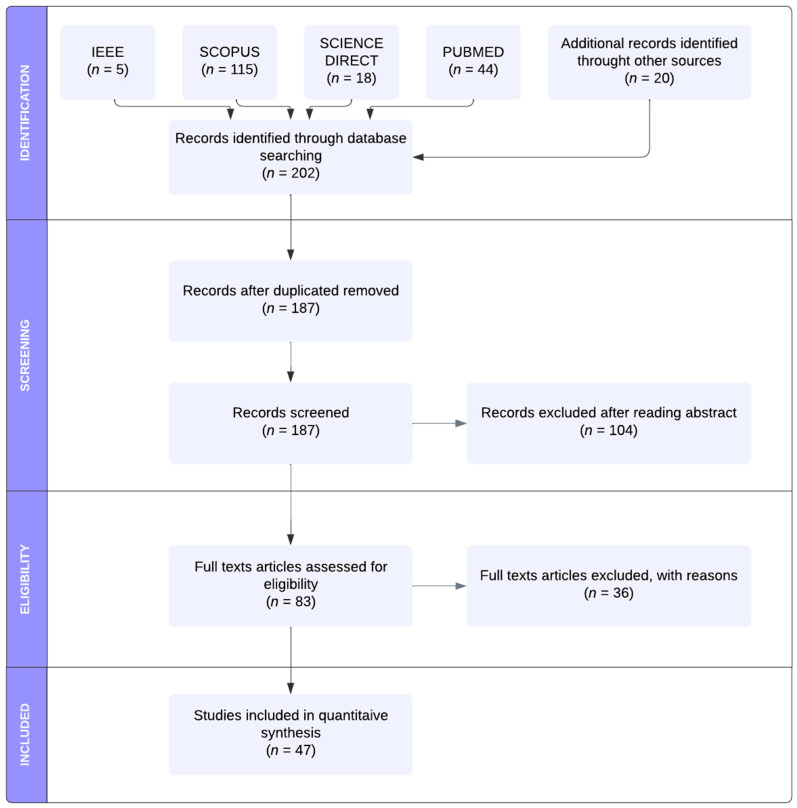
PRISMA workflow of the identification, screening, eligibility and inclusion of the studies in the systematic review.

A total of 202 search results were identified through database searching and additional sources. After removing all duplicates, 187 studies underwent title and abstract screening, and the inclusion criteria were examined. The full texts of 83 papers assessed for eligibility were carefully analyzed. Thirteen articles [16,17,18,19,20,21,22,23,24,25,26,27,28] were excluded due to the exclusion criteria (1), one contribution [29] due to the exclusion criteria (2), 17 scientific results [30,31,32,33,34,35,36,37,38,39,40,41,42,43,44,45,46] due to the exclusion criteria (3) and 5 [47,48,49,50,51] contributions due to the exclusion criteria (4). Finally, only 47 studies were included in the quantitative synthesis. Figure 1 illustrated the methodological approach used. In order to facilitate analysis and comparisons, all relevant HoloLens 2-based existing solutions and related system parameters were summarized and discussed in Section 4.

## 3. HoloLens 2 versus Other Commercially Available Optical See-Through Head-Mounted Displays

The epidemiological context of the coronavirus disease 2019 pandemic has had wide-reaching impacts on all segments and sectors of society, imposing severe restrictions on the individuals’ participation in daily living activities, mobility and transport, on access to education, services and healthcare. This scenario represented a unique chance to speed up the significant investments by technology companies, including Google, Apple, Microsoft, and Meta (Facebook), into eXtended Reality HMD technology [52]. Table 2 presents a basic overview of the relevant commercially available OST-HMDs: Google Glass 2 Enterprise Edition (Google, Mountain View, CA, USA) [53], HoloLens 1 [8] and 2 [9] (Microsoft, Redmond, WA, USA), Magic Leap 1 and 2 (Magic Leap, Plantation, FL, USA) [54,55]. The associated technical specifications are included (see for more information [52,56]).

### HoloLens First and Second Generation Comparison: A Detailed Study of Features, Functionalities and Performances

**Figure 2:**
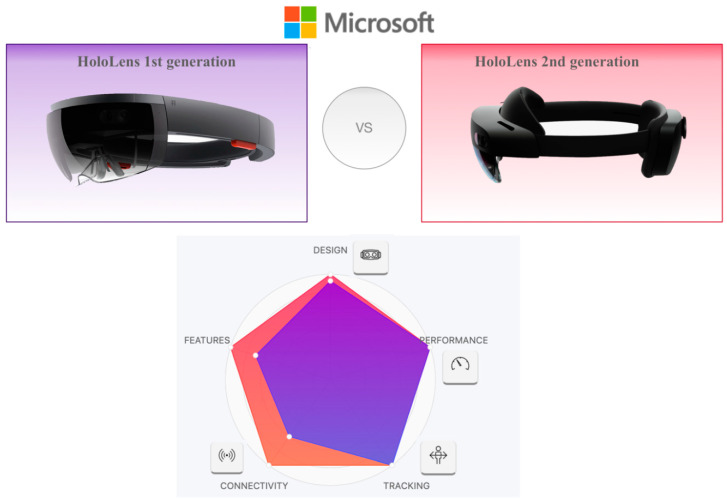
HoloLens’ first- and second-generation comparison.

This section illustrates a more detailed overview of HoloLens 2 technical specifications and examines the comparison with the previous version (HoloLens 1) [8] (Figure 2, Table 3), justifying the choice to focus our review only on the latest version. 

The first generation of HoloLens, released in 2016, attracted the consideration of the scientific and technological context because of its advanced playing methods and concepts.

However, the hardware and software limitations of the HoloLens version1, including its restricted field of view, limited battery life, and relatively heavy headset, prompted the company to introduce the HoloLens 2.

Indeed, in November 2019, Microsoft Corporation released the subsequent HoloLens 2, which is an upgrade in terms of hardware (enhanced field of view (52°), reduced weight (566 g) and improved battery life (3 h)) and software, compared with its predecessor. 

Considering the technical characteristics shown in Table 2 and Table 3, the Microsoft HoloLens 2 is the best head-mounted display headset on the market. It is a very elegant device, made with high-quality materials and offers, undisputedly, the best position tracking. The hand tracking works extraordinarily well and the 3D viewing is much more realistic (objects hardly wobble when moving and remain super stable). Moreover, to our knowledge, no other similar commercially available system has undergone the rigorous validation process of the HoloLens 2 [57].

## 4. MS HoloLens 2 Applications in Medical and Healthcare Context: Literature Results

In this section, several existing applications of MS HoloLens 2 in medical and healthcare context are carefully analyzed and illustrated. The main characteristics in terms of clinical sub-field applications, device functionalities provided to users, software/platform/framework used, as well as study validation are summarized in Table 4. 

The use of HoloLens 2 in a medical and healthcare context was analyzed by dividing contributions into the following sub-field applications: surgical navigation, AR-BCI (Brain-Computer Interface) systems integration and human computer interaction (HCI), gait analysis and rehabilitation, medical education and training/virtual teaching/tele-mentoring/tele-consulting and other applications.

### 4.1. Surgical Navigation

Most of the studies [58,59,60,61,62,63,64,65,66,67,68,69,70,71,72,73,74,75,76,77,78,79,80,81,82,83,84,85] in this review are focused on application of HoloLens 2 in surgical navigation in the operating room and in the emergency department. The use of the AR/MR based system in this context provided the user with computer-generated information superimposed to real-world environment and improved accuracy, safety and efficacy of surgical procedures [86]. The AR-based HoloLens 2 is mainly used as surgical aids aimed at the visualization of medical data, blood vessel search and targeting support for precise positioning of mechanical elements [86].

### 4.2. Human Computer Interaction and AR-BCI Systems Integration 

In recent years, the Brain-Computer Interface application has been growing rapidly, establishing itself as an emerging technology [87] tested in several scenarios such as rehabilitation [88], robotics [89], precision surgery and speech recognition. However, the usability of many professional brain-sensing equipment remains limited. Indeed, these systems remain expensive, bulky, uncomfortable to wear due to the gel applied to the electrodes and tethered, as well as prone to classification errors. Thus, the modern trend of the scientific community is directed to the use of BCI systems in association with other input modalities such as gaze trackers [90], or HMDs such as Virtual Reality (VR) [91] and Augmented Reality (AR) headsets [92,93]. 

Two research contributions [94,95] integrated the BCI and AR HMD systems within the same physical prototype. More specifically, in a first pilot study [94], the authors proposed a prototype which combines the Microsoft HoloLens 2 with an EEG BCI system based on covert visuospatial attention (CVSA)—a process of focusing attention on different regions of the visual field without overt eye movements. Fourteen participants were enrolled to test the system over the course of two days using a CVSA paradigm. In another study [95], considering the introduced clip-on solution for the AR-BCI integration, the authors designed a simple 3D game, which changed in real time according to the user’s state of attention measured via EEG and coupled the prototype with a real-time attention classifier. The results of these studies, though promising, needed to be considered preliminary due to the small number of participants (*n* = 14).

In addition to the described contributions [94,95], the work of Wolf et al. [96] fits into the human–computer interaction (HCI) field. More specifically, the authors analyzed hand-eye coordination in real-time to predict hand actions during target selection and thus giving the possibility to avoid users’ potential errors before they occur. In a first user study, the authors enrolled 10 participants and recorded them playing a memory card game, which involves frequent hand-eye coordination with little task-relevant information. In a second user study, considering a group of 12 participants, the real time effectiveness of the authors’ method to stop participants’ motions in time (i.e., before they reach and start manipulating a target), was evaluated. Despite this contribution’s limitation being represented by the small number of participants, the results demonstrated that the support of the implemented method was effective with a mean accuracy of 85.9%.

Another hot topic in virtual reality research is the use of embodied avatars (i.e., 3D models of human beings controlled by the user), or so-called full-body illusions, a promising tool able to enhance the user’s mental health. To complement this research, augmented reality is able to incorporate real elements, such as the therapist or the user’s real body, into therapeutic scenarios. Wolf et al. [97] presented a holographic AR mirror system based on an OST device and markerless body tracking to collect qualitative feedback regarding its user experience. Additionally, authors compared quantitative results in terms of presence, embodiment and body weight perception to similar systems using video see-through (VST), AR and VR. As results, the comparative evaluation between OST AR, VST AR, and VR revealed significant differences in relevant measures (lower feelings of presence and higher body weight of the generic avatar when using the OST AR system).

### 4.3. Gait Analysis and Rehabilitation

Augmented reality may be a technology solution for the assessment of gait and functional mobility metrics in clinical settings. Indeed, they provide interactive digital stimuli in the context of ecologically valid daily activities while allowing one to objectively quantify the movements of the user by using the inertial measurement units (IMUs). The project of Koop et al. [57] aimed to determine the equivalency of kinematic outcomes characterizing lower-extremity function derived from the HoloLens 2 and three-dimensional (3D) motion capture systems (MoCap). Kinematic data of sixty-six healthy adults were collected using the HoloLens 2 and MoCap while they completed two lower-extremity tasks: (1) continuous walking and (2) timed up-and-go (TUG). The authors demonstrated that the TUG metrics, including turn duration and velocity, were statistically equivalent between the two systems.

In the rehabilitation context, the developed technologies such as virtual and augmented reality can also enable gait and balance training outside the clinics. The study of Held et al. [98] aimed to investigate the manipulation of the gait pattern of persons who have had a stroke based on virtual augmentation during overground walking compared to walking without AR performance feedback. Subsequently, authors evaluated the usability of the AR feedback prototype in a chronic stroke subject with minor gait and balance impairments. The results provided the first evidence of gait adaptation during overground walking based on real-time feedback through visual and auditory augmentation.

### 4.4. Medical Education and Training/Virtual Teaching/Tele-Mentoring/Tele-Consulting

During the COVID-19 pandemic, undergraduate medical training was significantly restricted with the suspension of medical student clerkships onwards. Aiming to continue to deliver training for medical students, augmented reality has started to emerge as a medical education and training tool, allowing new and promising possibilities for visualization and interaction with digital content. 

Nine contributions [99,100,101,102,103,104,105,106,107] described the use of AR technology and the feasibility of using the HoloLens 2 headset to deliver remote bedside teaching or to enable 3D display for the facilitation of the learning process or in tele-mentoring and tele-consulting contexts.

Wolf et al. [99], for example, investigated the potential benefits of AR-based and step-by-step contextual instructions for ECMO cannulation training and compare them with the conventional training instructions regularly used at a university hospital. A comparative study between conventional and AR-based instructions for ECMO cannulation training was conducted with 21 medical students. The results demonstrated the high potential of AR instructions to improve ECMO cannulation training outcomes as a result of better information acquisition by participants during task execution.

Several studies [105,107] confirmed that the use of AR technology also enhanced the performance of tele-mentoring and teleconsulting systems in healthcare environments [105]. Tele-mentoring can be considered as an approach in which a mentor interactively guides a mentee at a different geographic location using a technological communication device.

Bui et al. [105] demonstrated the usability of AR technology in tele-mentoring clinical healthcare professionals in managing clinical scenarios. In a quasi-experimental study, four experienced health professionals and a minimum of 12 novice health practitioners were recruited for the roles of mentors and mentees, respectively. Each mentee wears the AR headset and performs a maximum of four different clinical scenarios (Acute Coronary Syndrome, Acute Myocardial Infarction, Pneumonia Severe Reaction to Antibiotics, and Hypoglycaemic Emergency) in a simulated learning environment. The role of a mentor, who stays in a separate room, is to use a laptop to provide the mentee remote instruction and guidance following the standard protocols related to each scenario. The mentors and mentees’ perception of the AR’s usability, the mentorship effectiveness, and the mentees’ self-confidence and skill performance were considered as outcome measures.

Bala et al. [106] presented a proof-of-concept study at a London teaching hospital using mixed reality (MR) technology (HoloLens 2™) to deliver a remote access teaching ward ward-round. The authors evaluated the feasibility, acceptability and effectiveness of this technology for educational purposes from the perspectives of students, faculty members and patients.

### 4.5. Other Applications

Four contributions [108,109,110,111] have been included in the “other applications” subgroup since, due to their characteristics, they cannot be configured as belonging to the subgroups mentioned. More specifically, in the study of Onishi et al. [108], the authors implemented a prototype system, named Gaze-Breath, in which gaze and breathing are integrated, using an MR headset and a thermal camera, respectively, for hands-free or intuitive inputs to control the cursor three-dimensionally and facilitate switching between pointing and selection. Johnson et al. [109] developed and preliminarily tested a radiotherapy system for patient posture correction and alignment using a mixed reality visualization. Kurazume et al. [110] presented a comparative study of two AR training systems for Humanitude dementia care, a multimodal comprehensive care methodology for patients with dementia. In this work, authors presented a new prototype called HEARTS 2 consisting of Microsoft HoloLens 2 as well as realistic and animated computer graphics (CG) models of older women. Finally, Matyash et al. [111] investigated accuracy measurement of HoloLens 2 inertial measurement units (IMUs) in medical environments. Indeed, the authors analyzed the accuracy and repeatability of the HoloLens 2 position finding to provide a quantitative measure of pose repeatability and deviation from a path while in motion.

**Table 4:** Summary of Microsoft HoloLens 2 applications in medical and healthcare context.

	Reference	Year	Aim of Study	Sub-Field Application	Methodology	Device Functionality	HoloLens 2 Natively Integrated and Other Software Used	Study Validation Type (*n* Participants)
**1**	**Wang et al. [58]**	2022	To establish a 3-dimensional visualization model of percutaneous nephrolithotomy, apply it to guiding intraoperative puncture in a mixed reality environment, and evaluate its accuracy and clinical value.	Surgical navigation	MR	3D visualization—Preoperative Planning	Vuforia Engine	3D group (Pz: *n* = 21) Control group (Pz: *n* = 40)
**2**	**Liu et al. [59]**	2021	To evaluate the use of MixR technology using OST-HMDs during TPED.		MR	To navigate the four procedures of marking, needle insertion, foraminoplasty, and positioning of the working sheath.	Mimics software version 20.0 (Interactive Medical Image Control System, Materialise, Leuven, Belgium) Scene Editing System (Midivi, Changzhou, China, https://www.midivi.cn (accessed on 16 February 2021) MixR system (Midivi, Changzhou, China)	Patients treated with MixR-assisted TPED through OST-HMDs (*n* = 44) were compared with matched patients treated with conventional TPED (*n* = 43).
**3**	**Eom et al. [60]**	2022	To present an AR assisted surgical guidance system that aims to improve the accuracy of catheter placement in ventriculostomy.		AR	AR-assisted surgical guidance	n.d.	On phantom model
**4**	**Kitagawa et al. [61]**	2022	To assess the safety and efficacy of laparoscopic cholecystectomy using a holography-guided navigation system as an intraoperative support image.		MR	Intraoperative imaging support	HoloeyesMD system (Holoeyes, Inc., Tokyo, Japan)	(Pz: *n* = 27)
**5**	**Doughty et al. [62]**	2022	To compare the perceptual accuracy of several visualization paradigms involving an adjacent monitor, or the Microsoft HoloLens 2 OST-HMD, in a targeted task and to assess the feasibility of displaying imaging-derived virtual models aligned with the injured porcine heart.		AR	Display of virtual models for guidance	Unity (https://unity.com/) (accessed on 22 September 2022). Eigen (https://eigen.tuxfamily.org/) (accessed on 22 September 2022) ArUco library	On MRI-based anatomical models, aligned with the surgically exposed heart in a motion-arrested open-chest porcine model.
**6**	**Torabinia et al. [63]**	2022	To present the use of a mixed reality headset (i.e., Microsoft HoloLens 2), as a tool for intra-procedural image-guidance during a mock myomectomy of an ex vivo animal uterus.		MR	Intra-procedural image guidance	Materialize Mimics Research software 21.0 SolidWorks 3D Viewer app	On custom-made uterine fibroid animal model
**7**	**Gsaxner et al. [64]**	2022	To present an AR-SNS for a commercial OST-HMD, the HoloLens 2.		AR	Tracking	n.d.	On 3D-printed patient Phantom
**8**	**Garciía-sevilla et al. [65]**	2022	To propose to use augmented reality to guide and verify PSIs placement in pelvic tumor resections		AR	Surgical guided Navigation	Unity platform (version 2019.3) Vuforia development kit	On plastic 3D-printed phantom
**9**	**Amiras et al. [66]**	2021	To present a simulator for CT-guided biopsies with haptic feedback using the HoloLens 2 and a bespoke software application.		AR	Real-time 3D mapping and tracking	HoloLens application (Microsoft Visual Studio 2019, the DirectX SDK, and the ChArUco implementation in OpenCV)	*n* = 16 users (CTR) trialled the application on 3D model of a torso
**10**	**Park et al. [67]**	2020	To describe the design of a 3D AR-assisted navigation system using the next-generation HoloLens 2 headset device.		AR	3D guidance to assist CT-guided targeting.	Unity 2019.2.21 Mixed Reality Toolkit Foundation 2.3.0 Vuforia 9.0.12	A prospective trial was performed assessing CT-guided lesion targeting on an abdominal phantom with and without AR guidance using HoloLens 2. (HC: *n* = 8)
**11**	**Benmahdjoub et al. [68]**	2021	To investigate the effect of instrument visualization/non-visualization on alignment tasks, and to compare it with virtual extensions approach which augments the realistic representation of the instrument with simple 3D objects.		AR	AR device	Unity	(HC: *n* = 18 volunteers)
**12**	**Benmahdjoub et al. [69]**	2022	To develop and assess a generic approach which aligns an AR device, such as the HoloLens 2, with existing navigation systems.		AR	AR device and tracking system	Vuforia 2020 MevisLab 2020	(HC: *n* = 10 volunteers)
**13**	**Farshad et al. [70]**	2021	To prove operator independent reliability and accuracy of both AR assisted pedicle screw navigation and AR assisted rod bending in a cadaver setting.		AR	AR-based surgical navigation	Mimics 19.0, Materialise NV, Leuven, Belgium Preoperative planning software (CASPA, University Hospital Balgrist, Zurich, Switzerland)	Experiments performed in human cadavers (HC: *n* = 2 biomedical engineers)
**14**	**Doughty et al. [71]**	2021	To present SurgeonAssist-Net: a lightweight framework making action-and-workflow-driven virtual assistance, for a set of predefined surgical tasks, accessible to commercially available OST-HMDs.		AR	Surgical Guidance	PyTorch	Online simulated surgical scenario and proprietary dataset for training the SurgeonAssist-Net framework
**15**	**Nagayo et al. [72]**	2022	To evaluate the effectiveness and usability of the suture training system for novices to learn a suture skill in open surgery, subcuticular interrupted suture, in comparison with the existing self-training system which uses instructional videos.		AR	AR training	n.d.	(HC: *n* = 43 medical students)
**16**	**Nagayo et al. [73]**	2021	To develop a new suture training system for open surgery that provides trainees with the three-dimensional information of exemplary procedures performed by experts and allows them to observe and imitate the procedures during self-practice.		AR	A 3D replication system of surgical procedures	Vuforia Engine (PTC, Inc., Boston, MA), Unity (Unity Technologies, San Francisco, CA), MRTK (Microsoft, Inc.).	(HC: *n* = 2)
**17**	von Haxthausen [74]	2021	**To propose an approach to automatically register a hologram to the according RWO.**		AR	Visual guidance	Unity 2019.4.15f1	To quantify the displacements between certain known positions between the virtual object and the RWO on torso phantom.
**18**	**Wierzbicki et al. [75]**	2022	To investigate the potential of a combination of 3D mixed-reality visualization of medical images using CarnaLife Holo system as a supporting tool for innovative, minimally invasive Surgery (MIS)/irreversible electroporation (IRA)/ microwave ablation (MWA)/for advanced gastrointestinal tumors.		MR	Mixed Reality Consultation	CarnaLife Holo	(Pz: *n* = 8)
**19**	**Brunzini et al. [76]**	2022	The proposed work aims to develop and test an AR application for different maxillofacial surgeries.		AR	AR surgical guides	Unity 2020.1.17f1 Visual Studio 2019	Preliminary laboratory validation (HC: *n* = 7)
**20**	**Thabit et al. [77]**	2022	To develop an AR-based system to visualize cranial sutures, and to assess the accuracy and usability of using AR-based navigation for surgical guidance in minimally invasive spring-assisted craniectomy.		AR	AR-based navigation	Vuforia (version 9.3, https://developer.vuforia.com/) (accessed on 22 September 2022)	(HC: *n* = 20)
**21**	**Cercenelli et al. [78]**	2022	To describe the AR-based protocol for assisting skin paddle harvesting in osteomyocutaneous fibular flap reconstructive procedure, usable both with a handheld device, such as a tablet, and with a HMD, such as Microsoft HoloLens 2 smart glasses.		AR		Unity 3D software (Unity Technologies, San Francisco, CA, USA) Vuforia Engine package, PTC, Inc., Boston, MA, USA	Experimental tests on phantom
**22**	**Felix et al. [79]**	2022	To determine the accuracy of pedicle screw placement using VisAR for open spine and MISS procedures.		AR	AR guidance	VisAR (Novarad, Provo, UT)	7 cadavers were instrumented with 124 thoracolumbar pedicle screws using VisAR augmented reality/guidance.
**23**	**Tu et al. [80]**	2021	To develop an augmented reality-based navigation system for distal interlocking of intramedullary nail using Microsoft HoloLens 2		AR	AR-based navigation system	Atamai Image Guided Surgery (AIGS) toolkit (https://github.com/dgobbi/AIGS) (accessed on 22 September 2022) Unity and C# Mixed Reality Toolkit (MRTK)	Phantom experiment (HC: *n* = 1 senior orthopedic surgeon)
**24**	**Zhou et al. [81]**	2022	To present a mixed reality surgical navigation system for glioma resection		MR	MR device (Surgical Navigation & Spatial Markers)	n.d.	Phantom experiments in an ideal environment in an operating room conducted by experienced surgeons (*n* = 20) Clinical trial (Pz: *n* = 16)
**25**	**Ivanov et al. [82]**	2021	To develop an approach that would allow surgeons to conduct operations using MR smart glasses MS HoloLens 2 on a large scale, reducing the preparation time required for the procedure and without having to create custom solutions for each patient.		MR	Visualization	Unity Vuforia SDK	3 clinical cases: - 2 median neck cysts (Pz: *n* = 1) - 1 branchial cyst (Pz: *n* = 1)
**26**	**Heinrich et al. [83]**	2022	To compare three state-of-the-art navigation concepts displayed by an optical see-through head-mounted display and a stereoscopic projection system.		AR	Visualization	Unity Vuforia AR SDK (PTC Inc, USA).	(HC: *n* *=* 24)
**27**	**Morita et al. [84]**	2022	To develop and assess the accuracy of a MR needle guidance application on smartglasses.		MR	MR needle guidance	Unity 2019.4.9 MR toolkit (MRTK v2.4.0, Microsoft) MR Needle Guide	Phantom experiment: the needle placement errors from 12 different entry points in a phantom by 7 operators (HC) were compared between the MR guidance and conventional methods
**28**	**Mitani et. al. [85]**	2021	To use a case-specific 3D hologram for tumor resection in otolaryngology, show the proof of concept.		MR	See-through head mount displays	ZIOSTATION Holoeyes XR system (Holoeyes Inc., Tokyo, Japan)	HDMs experience evaluation using 1uestionnaire: (HC: *n* *=* 18)
**29**	**Kosmyna et al. [95]**	2021	To integrate an EEG-BCI system with an AR headset, design a simple 3D game and couple the prototype with a real-time attention classifier.		AR	EEG-based BCI	Unity 3D	(HC: *n* *=* 14)
**30**	**Kosmyna et al. [94]**	2020	To propose a prototype which combines an existing AR headset, the Microsoft HoloLens 2, with EEG BCI system based on CVSA—a process of focusing attention on different regions of the visual field without overt eye movements.	Human computer interaction and AR-BCI systems integration	AR	EEG-based BCI	Unity 3D	(HC: *n* *=* 14)
**31**	**Wolf et al. [96]**	2021	To analyze hand-eye coordination in real-time to predict hand actions during target selection and warn users of potential errors before they occur.		AR	AR-Supported Manual Tasks	Unity’s 3D Game engine (2019.4.14f1) Mixed Reality Toolkit (MRTK 2.4.0).	Study 1: patterns in hand-eye coordination (HC: *n* = 11) Study 2: validating closed-loop user support (HC: *n* = 12)
**32**	**Wolf et al. [97]**	2022	To develop a holographic AR mirror system for investigating presence, avatar embodiment, and body weight perception in AR.		AR	Holographic AR mirror system	Unity 2020.3.11f1 LTS	(HC: *n* = 27)
**33**	**Koop et al. [57]**	2022	To determine whether the data derived from the HoloLens 2 characterizing lower extremity function during continuous walking and the TUG were equivalent to the outcomes derived using the gold standard MoCap system.		AR	Motion and biomechanical outcomes capture system	n.d.	(HC: *n* *=* 66)
**34**	**Held et al. [98]**	2020	(1) To investigate manipulation of the gait pattern of persons who have had a stroke based on virtual augmentation during overground walking compared to walking without AR performance feedback (2) To investigate the usability of the AR system.	Gait analysis and Rehabilitation	AR	AR parkour course visual system	n.d.	(Pz: *n* = 1)
**35**	**Wolf et al. [99]**	2021	The present study investigates the potential benefits of AR-based, contextual instructions for ECMO cannulation training as compared to instructions used during conventional training at a university hospital.		AR	AR guide system	Unity 3D Game Engine (Unity Technologies, San Francisco, California).	Comparison between conventional and AR-based instructions for ECMO cannulation training (HC: *n* = 21)
**36**	**Mill et al. [100]**	2021	To explore the feasibility of using a wearable headset to live stream teaching ward rounds to remotely based medical students.	Medical Training/Virtual teaching/Tele-mentoring and Tele-consulting systems	AR	Live streamed and remote teaching	Microsoft Teams	Live streamed teaching (HC: *n* *=* 53)
**37**	**Levy et al. [101]**	2021	To investigate the value and acceptability of using the Microsoft HoloLens 2 MR headset in a COVID-19 renal medicine ward.		MR			(HC: *n* = 16: 9 patients and 7 staff)
**38**	**Sivananthan et al. [102]**	2022	To assess the feasibility of using a MR headset to deliver remote bedside teaching.		MR			(HC: *n* *=* 24: 19 junior doctors and 4 specialist trainees)
**39**	**Rafi et al. [103]**	2021	To utilize a new AR technology (the Microsoft HoloLens 2) to deliver students a remote bedside teaching experience.		AR			(HC: *n* *=* 30: students)
**40**	**Dolega-** **Dolegowski et al. [104]**	2022	To describe the development of a Microsoft HoloLens 2-based application enabling 3D display of the internal anatomy of dental roots for facilitation of learning process.		AR	AR system	Autodesk Maya Unity software	(HC: *n* = 12: 6 Dental students 6 Dentists)
**41**	**Bui et al. [105]**	2022	To evaluate the usability of AR technology in tele-mentorship for managing clinical scenarios.		AR	Entirely hands-free operations, real-time annotations in 3D space, and document sharing	n.d.	(HC: *n* *=* 24: 4 mentors 12 mentees)
**42**	**Mentis et al. [107]**	2022	To introduce the use of AR HMD for remote instruction in healthcare and present the challenges author’s team has faced in achieving this application in two contexts: surgical telementoring and paramedic teleconsulting.		AR	Tele-mentoring and tele-consulting	Dynamics 365 Remote Assist https://dynamics.microsoft.com/it-it/mixed-reality/remote-assist/ (accessed on 22 September 2022) Microsoft Teams	n.d.
**43**	**Bala et al. [106]**	2021	To conduct a proof-of- Concept study at a hospital using mixed reality technology (HoloLens 2™) to deliver a remote access teaching ward round.		MR	Live-streaming, remote access, interactive teaching ward round for medical students.	Dynamics 365 Remote Assist https://dynamics.microsoft.com/it-it/mixed-reality/remote-assist/ (accessed on 22 September 2022) Microsoft Teams	(HC: *n* = 11) (Pz: *n* = 2)
**44**	**Onishi et al. [108]**	2022	To propose a combined gaze and breathing inputs system		MR	Gaze pointing function	Unity version 2020.2.2f1 Holographic Remoting Player 2.2.1	(HC: *n* = 10)
**45**	**Johnson et al. [109]**	2021	To develop and preliminarily test a radiotherapy system for patient posture correction and alignment using MixR visualization.	Other applications	MR	Live and visual reference system, enabling real-time feedback and on-line patient posture correction and alignment	Unity v2019.2.21f1 (Unity Technologies, San Francisco, CA) Mixed Realty Toolkit v2.4 (MRTK2.4) Visual Studio v2019 (Microsoft, Redmond, WA) 3D Slicer (www.slicer.org) (accessed on 22 September 2022) Vuforia SDK v9.2.8 https://developer.vuforia.com/ (accessed on 22 September 2022)	Preliminary estimation of registration accuracy (Phantom testing)
**46**	**Kurazume et al. [110]**	2022	To presents a new prototype (HEARTS 2) consisting of Microsoft HoloLens 2 as well as realistic and animated CG models of older women.		AR	AR training device	Mixed Reality Toolkit v2	4 experiments: (1) Psychological experiments to verify the improvements of HEARTS 2 vs HEARTS 1. (HC: *n* = 20) (2) Questionnaire survey regarding the usefulness of HEARTS 2 as a multimodal care training system. (HC: *n* = 20) (3) Questionnaire survey for medical professionals regarding the usefulness of HEARTS 2 as a multimodal care training system. (HC: *n* = 6, 5 physicians and 1 nurse) (4) Preliminary training experiments regarding multimodal care using HEARTS 2. (HC: *n* = 4)
**47**	**Matyash et al. [111]**	2021	To investigate the accuracy and precision of the HoloLens 2 position finding capabilities, quantify the pose repeatability and the deviation of the device from a known trajectory.		AR	Position and motion tracking	Unity Visual Studio 2019	Measurements of pose repeatability and path deviation during motion.

n.d.: not defined; 3D: three dimensional; AR: augmented reality; BCI: Brain Computer Interface; EEG: electroencephalogram MR: mixed reality; OST-HMD: optical see-through head-mounted display; CVSA: covert visuospatial attention; CG: Computer Graphics; SNS: Surgical navigation systems; PSIs: Patient-specific instruments; HC: healthy control; RWO: real-world objects; TPED: transforaminal percutaneous endoscopic discectomy; Pz: patients; MISS: minimally invasive spine surgery.

In light of the results summarized in Table 4, this review allows us to present a thorough examination of the different studies conducted since 2019, focusing on HoloLens 2 applications and to analyze the current status of publications by year, typology of publications (articles or conference proceedings), sub-field applications, types of visualization technologies and device functionality.

**Figure 3:**
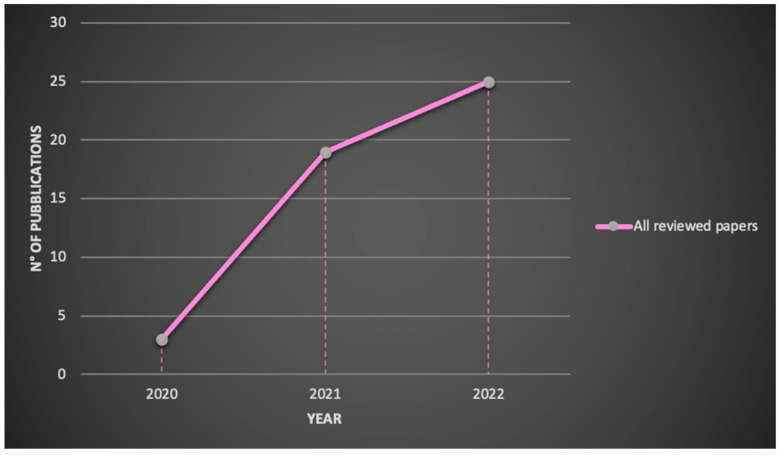
Reviewed publications related to HoloLens 2 research by year.

The number of publications on the Microsoft^®^ HoloLens 2 application in a medical and healthcare context is shown in Figure 3. 

Starting from the year following the release of the second-generation product, the demand for HoloLens 2 has increased exponentially in medical sector until today and the research is expected to expand further in the future. Indeed, in 2020 the number of publications was 3, increasing to 19 in 2021 and to 25 in 2022.

**Figure 4:**
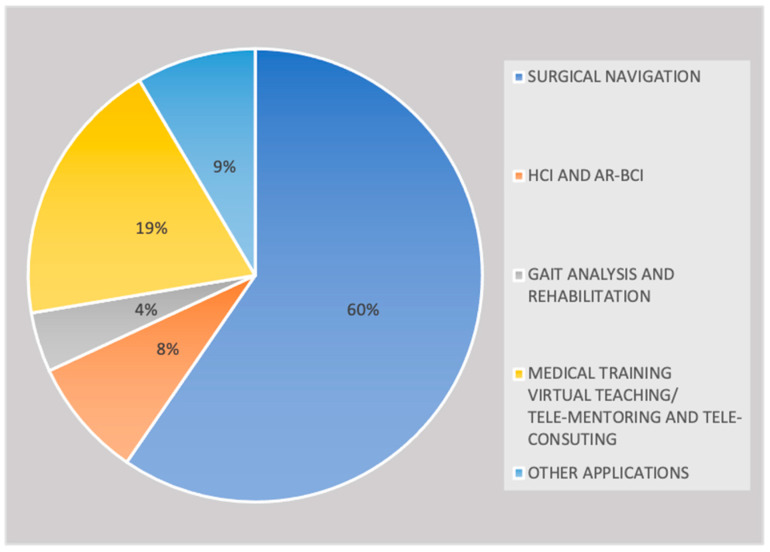
Reviewed publications related to HoloLens 2 research by sub-field applications.

In addition, the use of HoloLens 2 in a medical and healthcare context was analyzed by dividing contributions into the following sub-field applications: surgical navigation, AR-BCI systems integration and human computer interaction, gait analysis and rehabilitation, medical education and training/virtual teaching/tele-mentoring/tele-consulting and other applications. Figure 4 illustrates that surgical navigation represents the most common application (60%, *n* = 28) of HoloLens 2 and that also in medical training /virtual teaching/tele-mentoring and tele-consulting contexts, the use of this methodology is increasing considerably (19%, *n* = 9).

Despite the enormous potential of augmented reality in gait analysis and rehabilitation as well as in a brain computer interface, few studies have been published so far (4%, *n* = 2 and 8%, *n* = 4).

**Figure 5:**
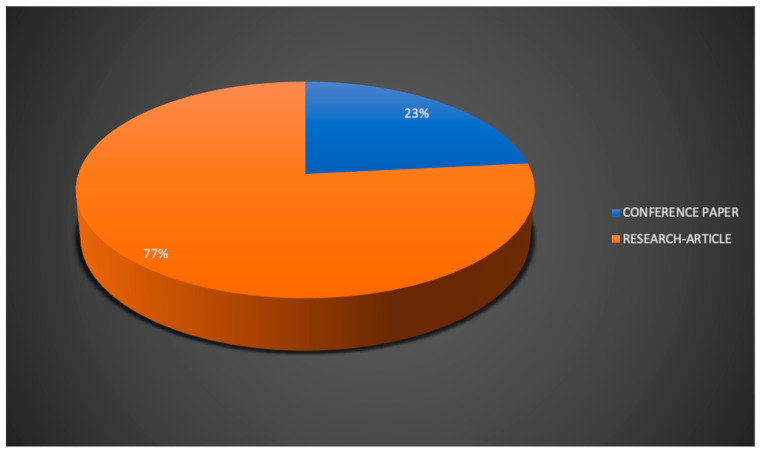
Reviewed publications related to HoloLens 2 research by type of publication.

Concerning the type of publication, most of the reviewed papers were research articles (77%, *n* = 36), while a smaller percentage (23%, *n* = 11) was composed of conference proceedings (Figure 5).

**Figure 6:**
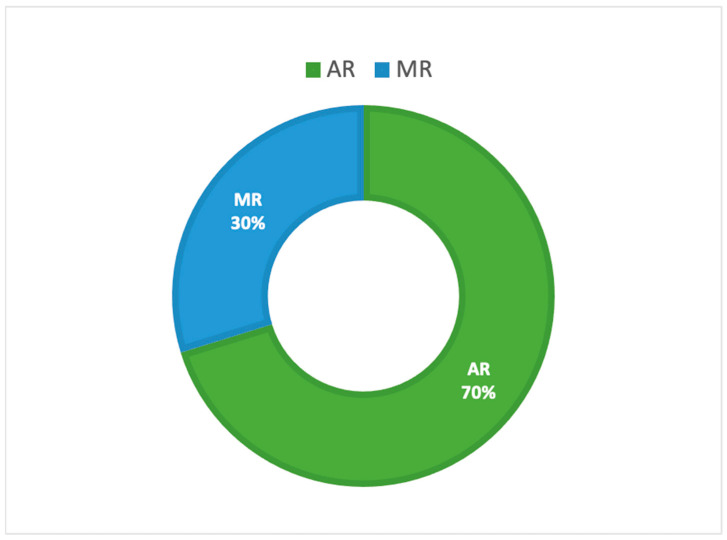
Reviewed publications related to HoloLens 2 research by types of visualization technologies.

Analyzing our review results in terms of types of visualization technologies (Figure 6), the two types of approaches, AR and MR, were used for applications in a medical and healthcare context. More specifically, AR application was the most common, as evidenced by its use in = 33 research papers (70%), while MR was present in only 14 contributions (30%).

The results of our review demonstrate that in most of the works, information relating to study validation are often missing or poorly described. However, we believe it is appropriate to report, where available, some details on how the HoloLens 2 performance assessment was achieved.

## 5. Discussion

Our paper aims to present the state-of-the-art applications of the Microsoft^®^ HoloLens 2 in a medical and healthcare context. This study reviewed academic papers that proved the applicability and feasibility of HoloLens 2 in a medical and healthcare context since 2019.

Although important benefits have already been identified from using the HoloLens 2 for medical use and vast improvements in eXtended Reality technologies have been achieved, some issues still need to be considered and resolved.

Indeed, despite augmented reality having demonstrated a great potential in clinical and healthcare context, the execution has been a little disappointing.

Some of the main technical limitations of today’s generations of AR headsets are the limited field of view in which overlays can be displayed and the limited battery life. In addition to the standard AR display-related performance, other characteristics such as ergonomics and mechanical design as well as the total weight of the headset play a crucial role in facilitating the acceptance of the AR HMD. Indeed, the HMD design itself can be the reason of a bad user experience due to limited FoV and the extra weight on a user’s head. This aspect is becoming less of an issue thanks to the rapid improvements in HMD design [112].

A recently published work [113] describes the ergonomic requirements that impact the mechanical design of the AR HMDs, suggesting the possible innovative solutions and how these solutions have been used to implement the AR headset in a clinical context.

HoloLens 2 is characterized by improved comfort compared with the alternatives. Indeed, it is lighter to wear, it is easier to get on and off too, it presents a more balanced center of gravity, and an improved heat management, meaning that the HoloLens 2 will fit a greater number of head shapes and sizes more comfortably [114]. More in detail, this device is incredibly balanced, thanks to the fact that the battery is on the rear and the display is on the front. The carefully studied weight distribution makes it so that the device does not touch the nose of the user, but it rests on his/her forehead.

Despite the ergonomic and design improvements, many people still report experiencing cybersickness symptoms from the AR HMD use [115,116,117].

Cybersickness is a term that identifies the cluster of symptoms that a user experiences during or after exposure to an immersive environment [118]. A physiological response to an unusual sensory stimulus, similar to motion sickness, characterizes this phenomenon, whose incidence and degree of intensity vary based on the exposure duration and nature of the virtual content and display technology [116]. The integration of the real and virtual environments in AR devices should reduce the adverse health effects that the user experienced in VR applications, such as blurred vision, disorientation and cybersickness [119].

In HoloLens devices, the precise 3D models presented, as well as the hands-free nature and ability to manipulate holographic images in real space, make this technology suitable for use in health science and medical education. In addition, based on scientific results [117], eyestrain seems to be the most common and prominent symptom caused by using the HoloLens, but it appeared less frequent and milder than in comparable virtual reality simulators. Innovative research on how to alleviate these symptoms would certainly be beneficial for allowing the prolonged use of these devices.

Another element of particular interest is to investigate how older adults interacted with this increasingly prevalent form of consumer immersive eXtended Reality technology to support Enhanced Activities of Daily Living (EADLs) and whether older adults’ psychological perception of technology is different compared to younger adults. Despite it being scientifically proven that older adults are more sensitive to simulator or cybersickness, the relationship between age effect and cybersickness may be complex [120,121].

Gender can be considered an additional relevant factor in the evaluation of eXtended Reality experiences. Although it has been proven that as older women may be especially susceptible to simulator/cybersickness, gender effects in the literature are inconsistent (for review, see [114]).

Another key point regarding the use of HoloLens in combination with XR modalities concerns the way to improve the holographic experience for the user, providing him/her with the haptic feedback. This term identifies the condition under which whenever the user touches (virtually) any projected hologram, the user has a sensation of a physical touch, depending on the inclinations of the object and the fingers of the user. The HoloLens 2 headset allows one to create a tactile virtual world for the users. Indeed, within holograms, audio effects give users the sense of pressing a button or flipping a switch. 

In addition, it has a higher hand tracking precision compared to other windows-based devices, and its development suite created for XR (Interhaptics [122]) provides solid hand interaction performances and will optimize the immersive experience for the end-user.

The lack of guidelines, protocols and standardization in using HMD devices as well as poor information on study validation represent the most critical aspects in describing the feasibility and applicability of the HoloLens 2.

One potential direction for this research field is represented by the machine learning (ML) applications. Indeed, considering the increasing trend of machine learning adoptions in all medical sectors, and in particular in medical image processing, such methods are likely to be applied to OST-HMD solutions [56]. 

The wealth of information that all OST-HMD systems record (video images, gesture-based interaction data, eye tracking and generated surface meshes) could provide rich training data for ML algorithms. Especially Neural Networks (NNs), the most important ML method for image recognition, translation, speech detection, spelling correction, and many more applications, would offer great opportunities on AR/MR devices.

More specifically, the HoloLens 2 presents the built-in gaze tracking, thus offering innovative HCI applications that still need to be explored, especially in a surgical setup. The creation of available data bases with relevant user data, which can then serve as inputs for ML algorithms, could represent a fundamental step in terms of accelerating ML research in the surgical field.

In conclusion, the results provided in this review could highlight the potential and limitations of the HoloLens 2-based innovative solutions and bring focus to emerging research topics, such as telemedicine, remote control and motor rehabilitation.

Despite the aforementioned limitations, the integration of this technology into clinical workflows, when properly developed and validated, could bring significant benefits such as improved outcomes and reduced cost [123], as well as decreasing the time physicians spend in health parameter recording.

## 6. Conclusions

This systematic review provides state-of-the-art applications of the MS HoloLens 2 in the medical and healthcare scenario. It presents a thorough examination of the different studies conducted since 2019, focusing on HoloLens 2 clinical sub-field applications, device functionalities provided to users, software/platform/framework used, as well as the study validation. Considering the huge potential application of this technology, also demonstrated in the pandemic context of COVID-19, this systematic literature review aims to prove the feasibility and applicability of HoloLens 2 in a medical and healthcare context as well as to highlight the limitations in the use of this innovative approach and bring focus to emerging research topics, such as telemedicine, remote control and motor rehabilitation.

## Figures and Tables

**Table 1:** Databases used for this review.

Database Name	URL	Date Access
Pubmed	https://pubmed.ncbi.nlm.nih.gov/	19 April 2022
IEEEXplore	https://www.ieee.org/	19 April 2022
Science Direct	https://www.sciencedirect.com/	19 April 2022
Scopus	https://www.scopus.com/	19 April 2022

**Table 2:** Basic technical specifications for commercially available optical see-through head-mounted displays.

	Google Glass 2	HoloLens 1	HoloLens 2	Magic Leap 1	Magic Leap 2
Specifications					
Release Date	2019	2016	2019	2018	2022
Price	$999	$3000	$3500	$2295	$3299
Status	Available	Discontinued	Available	Available	Upcoming
Design	Glasses-like	Hat-like	Hat-like	Glasses-like	Glasses-like
Weight	46 g	579 g	566 g	345 g	260 g
Battery life	8-h	2.5-h	3-h	3/3.5-h	3.5-h continuous use 7-h sleep mode
Interaction	Touchpad	Head, hand, voice	Head, hand, voice	Controller	Eye, controller
Eye Tracking	No	No	Yes	Yes	Yes
Computing	On-board	On-board	On-board	On-board	External pad
Field of View	30° diagonal	30 × 17.5°	43 × 29°	40 × 30°	44 × 53°
Focal Planes	Single Fixed	Single Fixed	Single Fixed	Two Fixed	Single Fixed
Optics	Beam Spitter	Waveguide	Waveguide	Waveguide	Waveguide
SLAM	6 DoF	6 DoF	6 DoF	6 DoF	6 DoF
**PRO**	Super lightweight and very unobtrusive; battery life	Comfortable; easy to use; support for Microsoft platforms	Comfortable; easy to use; very elegant device high-quality materials; navigation with hand gestures and voice; excellent positional tracking	Large FoV	Largest FoV
**CONS**	Intended for developers, only a few applications available natively	Small field of view; text can be difficult to read	Battery life; less suitable for industry	Price; battery life	Less suitable for use in heavy industry

SLAM: simultaneous localization and mapping capabilities; DoF: degrees of freedom.

**Table 3:** HoloLens 2 specifications compared to the first-generation HoloLens.

			HoloLens 2	HoloLens 1
**COMPUTE** **SPECIFICATIONS**		**CPU Model**	Qualcomm Snapdragon 850 Compute Platfom	Intel Atom x5-Z8100P @ 1.04 Ghz
		**Core Architecture**	ARM Cortex-A75	Intel Airmont
		**Logical CPU Cores**	8	4
		**Instruction Set**	ARMv8	32-bit X86
		**Memory**	4 GB LPDDR4× DRAM	1 GB LPDDR3
		**Storage**	64 GB UFS 2.1	64 GB
**HPU**		**Model**	2nd generation custom-built holo-graphic processing unit	1st generation custom-built holographic processing unit
		**HPU Memory**	Not specified	1 GB LPDDR3 RAM
**WIRELESS** **CONNECTIVITY**		**Wifi**	WiFi 5 (802.11ac 2 × 2)	WiFi 5 (802.11ac)
		**Bluetooth**	Bluetooth LE 5.0	Bluetooth 4.1 + BLE
		**USB**	USB Type-C	Micro USB 2.0
**DISPLAY**		**Optics**	See-through holographic Lenses (waveguides)	See-through holographic lenses (waveguides)
		**Resolution**	2k 3:2 light engines (screen aspect ratio)	2 HD 16:9 light engines (screen aspect ratio)
		**Holographic density**	>2.5k radiants (light points per radian)	2.5k radiants (light points per radian)
		**Eye-based rendering**	Display optimization for 3D eye position	Automatic pupillary distance calibration
		**Visible FoV**	43° horizontal 29° vertical 52° diagonal	30° horizontal 17° vertical
**AUDIO**		**Microphone array**	5 channels	4 channels
**SENSORS**	**CAMERA**	**Resolution**	8-MP stills,	2.4 MP (2048 × 1152)
		**Video Resolution**	1080 p30	1.1 MP (1408 × 792)
		**Video Speed**	24 fps	30 fps
	**IMU**	**Accelerometer, gyroscope, magnetometer**	1	1
	**AUDIO**	**Speakers**	Built-in, spatial audio	Built-in, spatial audio

FoV: Field of View; fps: frames per second.

## Data Availability

Not applicable.
